# Innovations in monoclonal antibody-based multipurpose prevention technology (MPT) for the prevention of sexually transmitted infections and unintended pregnancy

**DOI:** 10.3389/frph.2023.1337479

**Published:** 2024-01-09

**Authors:** Sarah Dohadwala, Matthew T. Geib, Joseph A. Politch, Deborah J. Anderson

**Affiliations:** ^1^ Department of Virology, Immunology and Microbiology, Boston University Chobanian & Avedisian School of Medicine, Boston, MA, United States; ^2^ Department of Material Science and Engineering, Boston University, Boston, MA, United States; ^3^ Department of Medicine, Boston University Chobanian & Avedisian School of Medicine, Boston, MA, United States

**Keywords:** multipurpose prevention technology, monoclonal antibodies, STI, HIV, contraception

## Abstract

Monoclonal antibodies (mAbs) are currently being produced for a number of clinical applications including contraception and the prevention of sexually transmitted infections (STIs). Combinations of contraceptive and anti-STI mAbs, including antibodies against HIV-1 and HSV-2, provide a powerful and flexible approach for highly potent and specific multipurpose prevention technology (MPT) products with desirable efficacy, safety and pharmacokinetic profiles. MAbs can be administered systemically by injection, or mucosally via topical products (e.g., films, gels, rings) which can be tailored for vaginal, penile or rectal administration to address the needs of different populations. The MPT field has faced challenges with safety, efficacy, production and cost. Here, we review the state-of-the-art of mAb MPTs that tackle these challenges with innovative strategies in mAb engineering, manufacturing, and delivery that could usher in a new generation of safe, efficacious, cost-effective, and scalable mAb MPTs.

## The case for MAb MPTs

The global prevalence of STIs and unplanned pregnancies remains unacceptably high despite the availability of prevention strategies to curtail many such adverse reproductive health outcomes. Over 400 million new cases of STIs and 121 million unplanned pregnancies occur every year. Common bacterial and parasitic STIs (e.g., gonorrhea, syphilis, chlamydia, trichomoniasis) are curable with existing single dose regimens of antibiotics, yet these STIs continue to infect over 350 million people a year due to a large percentage of untreated, asymptomatic, and recurrent infections, and drug resistance. Effective vaccines have been approved for three viral STIs: Human Papilloma Virus (HPV), Hepatitis A and Hepatitis B, yet vaccine coverage remains suboptimal in many regions (1). No vaccines or cures exist yet for two other highly pathogenic viral STI's, herpes simplex virus (HSV) types-1 and -2 and the human immunodeficiency virus type 1 (HIV-1). Currently over 700 million people worldwide are infected with genital HSV and 39 million people are living with HIV. Antiviral drugs can reduce viral load and transmission, but sexual transmission of these viruses is still common: 24 million new cases of genital HSV and 1.3 million cases of HIV are acquired annually (2, 3). Despite the availability of diverse contraceptive methods, many are unacceptable or unavailable to a large percentage of the world's population and as a consequence nearly 50% of pregnancies are unintended (4). MPTs are methods that provide dual protection against HIV, other STIs and/or unintended pregnancies. Recent surveys indicate a high level of user preference for MPT products (5), yet the only currently approved MPT product, the condom, is used by only a small percentage of sexually active adults (6). For these reasons, new more acceptable MPT methods are urgently needed to combat the current reproductive syndemic of STIs and unintended pregnancy.

**Figure 1:**
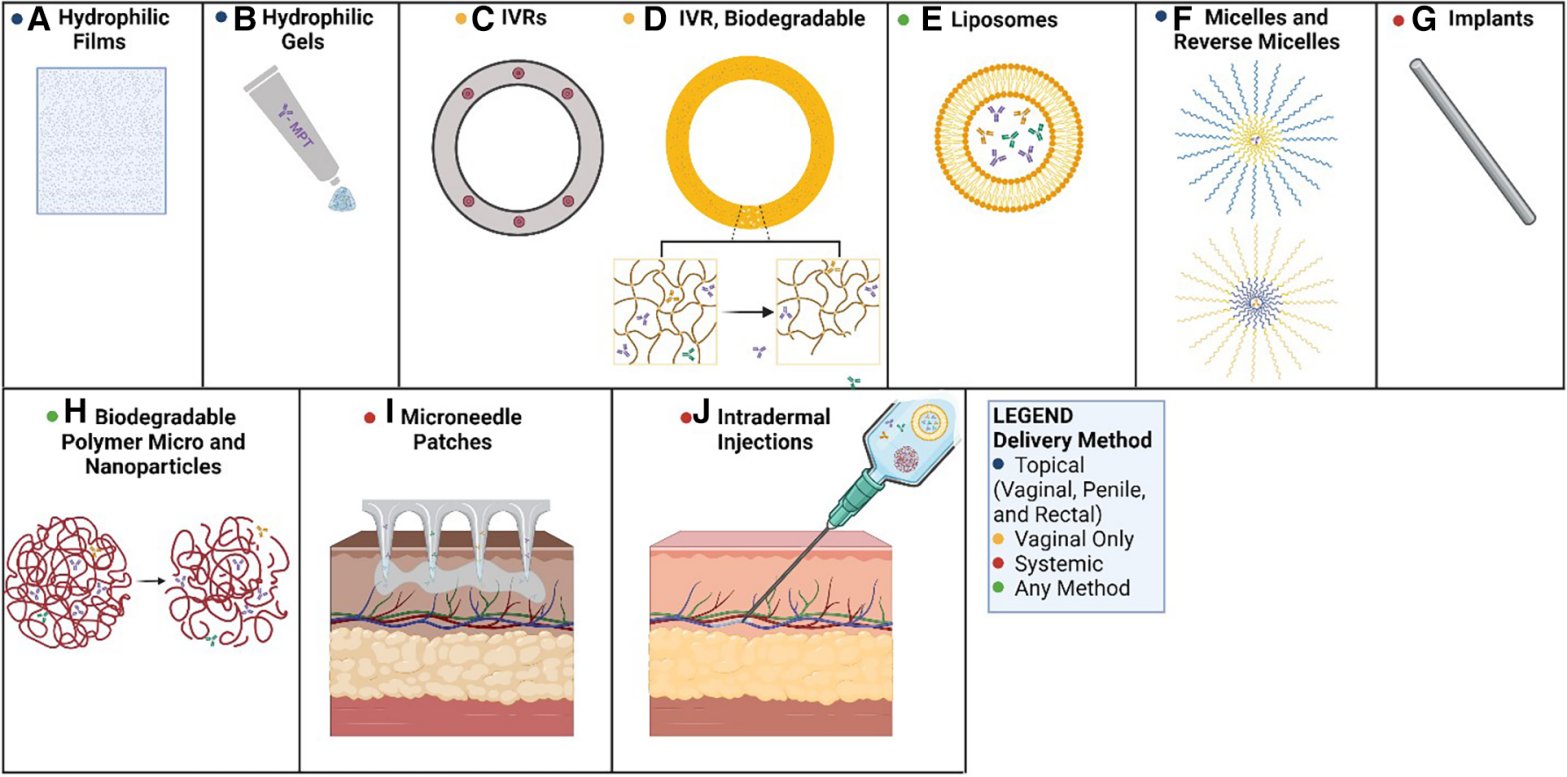
Methods of mAb-based MPT delivery. Potential routes for the delivery of mAb-based MPTs: (**A**) PVA-based films dissolve in the vagina, releasing mAbs; (**B**) hydrophilic gels can be applied to the penis, the rectum, or the vagina as a topical route of delivery; (**C**) PLGA or PCL pods erode releasing mAbs over time; (**D**) biodegradable polyurethane rings and implants erode, releasing antibodies over time; (**E**) liposomes can be delivered in hydrophilic suspensions or gels for topical applications or systemic delivery; (**F**) micelles can be used in hydrophilic solutions (reverse micelles in hydrophobic solutions) topically as creams; (**G**) biodegradable implants could deliver antibodies systemically as the matrix erodes; (**H**) biodegradable micro and nanoparticles erode allowing antibodies to diffuse from the matrix; (**I**) microneedle transdermal patches applied to the skin can systemically deliver mAbs over time; (**J**) intradermal injections can deliver a bolus dose of mAbs, or a solution of delivery vehicles for extended circulation time (e.g., nanoparticles, liposomes). Created with BioRender.com.

In this review we advocate for the use of monoclonal antibodies (mAbs) as MPTs. MAbs have emerged in recent years as invaluable drugs for the treatment of a variety of clinical conditions. They are potent and highly specific, have an excellent safety profile, and can be used in various combinations to simultaneously recognize and inactivate STIs and contraceptive targets such as sperm. Advanced antibody engineering is being used to develop more effective mAbs, and new mAb production platforms are addressing cost and supply issues. Different delivery methods enable systemic or local applications, and short-term (on-demand) or long-term protection (Figure 1). The timing is right for the advancement of mAb-based MPTs.

## Use of mAbs to prevent infectious diseases

Passive immunization, the administration of antibodies to people for the prevention and treatment of diseases, has been practiced for over a century (7). Early use of antisera was associated with severe side effects, but the invention of mAbs in 1975 and the introduction of enhanced human antibody cloning, screening and production techniques in the early 21st century gave rise to a new era of safer passive immunization. In 1986, the first mAb clinical product, muromonab-CD3 (OKT3), was approved for the treatment and prevention of acute rejection after solid organ transplantation (8). To date at least 137 mAbs have been approved by the FDA and/or EU for clinical use (9), and over 570 novel mAb products are in early commercial development (10). At present only two mAbs have been approved by the FDA for the treatment of infectious diseases, specifically anthrax and respiratory syncytial virus (RSV). Other mAbs targeting dangerous pathogens such as Ebola and SARS CoV-2 have received emergency use authorization (9). Several mAbs against STI pathogens (e.g., HIV-1 and HSV-2) are currently undergoing clinical trials. Academic centers and global non-profit consortia have been leading the way in mAb discovery for infectious diseases.

## MAbs against STI pathogens

### Human immunodeficiency virus type 1 (HIV)

The WHO has identified mAbs as an important approach for HIV prevention (11). Anti-HIV mAbs are commonly derived from HIV-infected individuals that have developed “broadly neutralizing antibodies (bnAbs)”, potent antibodies that are capable of neutralizing a wide variety of HIV strains. BnAbs develop over time from intense affinity maturation in the germinal centers. Over 500 monoclonal HIV bnAbs have been characterized to date (12). BnAbs primarily block HIV infection through viral neutralization; secondary mechanisms include complement-dependent cytotoxicity (CDC), antibody-dependent cellular phagocytosis (ADCP), and antibody-dependent cellular cytotoxicity (ADCC) (13). Many HIV bnAbs are currently undergoing testing in clinical trials for HIV treatment and prevention (Table 1A).

**Table 1:** Published clinical trials of HIV and HSV mAbs.

Name	Description	Stage of development
A. HIV BnAbs		
VRC01 (7, 14–17)	CD4-binding site (CD4-BS)	Phase I and IIb clinical trials
VRC01LS (18)	LS variant-extended serum half-life	Phase 1 clinical trial
VRC07-523LS (19–21)	CD4-BS	Phase I clinical trials
3BNC117 (22, 23)	CD4-BS	Phase I clinical trials
PGT121 (24)	V3 Glycan	Phase I clinical trial
10-1074 (25)	V3 Glycan	Phase I clinical trial
CAP256V2LS (21)	V2 Glycan	Phase I clinical trial
PGDM1400 (26)	V2 Glycan	Phase I clinical trial
Combination mAbs		
3BNC117	CD4-BS	Phase Ib clinical trial
10-1074 (27)	V3 Glycan	
3BNC117	CD4-BS	Phase I clinical trial
10-1074 (23, 28, 29)	V3 Glycan	
VRC07-523LS	CD4-BS	Phase I clinical trial
PGT121 (20)	V3 Glycan	
PGDM1400	V2 Glycan	Phase I clinical trial
PGT121	V3 Glycan	
VRC07-523LS (26)	CD4-BS	
CAP256V2LS	V2 Glycan	Phase I clinical trial
VRC07-523LS (21)	CD4-BS	
B. HSV mAbs		
HDIT101 (30) (h2c)	Humanized mAb, recognizes conserved epitope in glycoprotein B present on HSV-1 and HSV-2 and virus-infected cells.	Phase 1 clinical trial
UB-621/E317 (31)	A gD-specific human IgG1 mAb that can neutralize HSV-1 and HSV-2. UB-621 is the clinical grade product of E317.	Phase 1 clinical trial
		Phase 2 clinical trial
		Phase 2 clinical trial
		Phase 2 clinical trial
HSV8 (7)	Glycoprotein D-specific human IgG1 with the capability of neutralizing HSV-1 and HSV-2.	Phase 1 clinical trial

The first HIV mAbs to be tested in clinical trials were in a product containing three HIV bnAbs: 2F5, 4E10 and 2G12. Systemic intravenous infusion of the product and topical administration of a vaginal gel containing these antibodies (MABGEL) was determined to be safe (32, 33). The more potent HIV bnAb, VRC01, has undergone extensive investigation in clinical trials. The first studies demonstrated safety and pharmacokinetics following intravenous infusion (14). VRC01 was recently tested in two parallel phase 2b trials for HIV prevention involving thousands of patients (34). MAb infusions up to 30 µg/kg did not reduce the overall risk of acquiring an HIV infection due to the prevalence of VRC01-resistant strains where the trials were conducted. However, a subanalysis of the data revealed that VRC01 infusion provided a 75% prevention efficacy against HIV strains that were susceptible to the neutralizing effects of the antibody, providing proof of concept for the approach (34). VRC01 was administered in a topical vaginal film, MB66, to female volunteers. Single and multiple daily doses of the MB66 film were safe and vaginal lavages showed efficacy in ex vivo viral neutralization assays up to 24 h after dose administration (7). A series of new HIV mAbs that recognize diverse HIV epitopes have recently been tested in phase 1 clinical trials including: VRC07 and 3BNC117 (CD4 binding site), CAP256V2LS and PGDM1400 (V2 glycan), and PGT 121 and 10-1074 (V3 glycan) (35). Combinations of anti-HIV antibodies are better equipped than single antibodies to prevent the development of resistance by targeting non-overlapping epitopes. Thus, further trials involving two or three bnAb combinations are underway. In addition, bi- and tri- specific antibodies that target multiple epitopes of HIV Env are being tested (36).

### Herpes Simplex Virus (HSV)

At least 50 mAbs against HSV have been produced and shown to have antiviral effects *in vitro* and in animal HSV infection models [reviewed in Backes et al. (37)]. Three anti-HSV mAbs, 2C (HDIT101), E317 (UB-621) and HSV8, have progressed to human clinical trials (Table 1B).

2C is a humanized mAb that recognizes a conserved region of glycoprotein-B on the surface of HSV-1 and 2. It was determined to be safe after intravenous administration of escalating doses (maximum dose: 12,150 mg) to healthy volunteers (30). This antibody is currently being tested in phase 2 clinical trials for the treatment of orolabial HSV-1 lesions (NCT04539483), and anogenital HSV-2 lesions (NCT04165122).

E317 is an HSV glycoprotein-D specific human IgG1 mAb that can neutralize both HSV-1 and -2. The clinical grade of this mAb, UB-621, was recently determined to be safe and well tolerated in a phase 1 single dose (100 mg/ml) study in healthy volunteers; it is currently being tested in clinical trials for the prevention of orolabial and genital disease (37) (NCT02346760, NCT03595995, NCT04714060, NCT04979975). HSV8 is a human IgG1 mAb that recognizes HSV glycoprotein-D. A vaginal film, MB66, containing HSV8 and the anti-HIV mAb VRC01, was recently tested in a phase 1 clinical trial; no serious adverse events were reported, and antiviral neutralizing activity (both HIV and HSV) was detected in vaginal secretions from the women up to 24 h after treatment (7).

### Other STIs

A large number of mAbs have been manufactured against other STI pathogens and are currently used for research and diagnostic applications. To our knowledge, none has been tested to date in clinical trials, although mAbs against *Neisseria gonorrhoeae* may be advanced into the clinical arena soon due to the emergence of multidrug-resistant strains of Neisseria. The mAb 2CT against *N. gonorrhoeae* appears poised to enter clinical trials (38).

## MAbs as immunocontraception

The field of immunocontraception began in the early 1900s with the discovery that sperm can elicit immune responses in animals and humans, and observations that many infertile men and women have antisperm antibodies (39). International agencies including the World Health Organization (40) and the Population Council previously had contraceptive vaccine programs directed at eliciting immune responses to inhibit sperm, oocytes, placenta, and endocrine processes. In the 1980s and 90s, clinical trials were conducted to test the safety and efficacy of a β-HCG subunit contraceptive vaccine targeting human chorionic gonadotropin (hCG), a hormone secreted by the blastocyst early in pregnancy to ensure uterine receptivity. The hCG vaccine trials demonstrated safety, but antibody levels were highly variable indicating that some women would not be protected (41). Steps were underway to improve efficacy and to develop other contraceptive vaccines (e.g., against sperm), when the program was terminated in 1997 following concerns about safety, reversibility and potential for misuse (42).

Today, the immunocontraception approach is undergoing a renaissance due to breakthroughs in bioengineering and the ability to produce clinical grade human mAbs. Passive immunization bypasses the concerns raised with contraceptive vaccines which require active immunization: passive immunization is safe and reversible, and the dose and site of administration can be precisely controlled (43).

One promising immunocontraception candidate is an antisperm mAb, “Human Contraception Antibody (HCA)”, that recognizes a sperm surface carbohydrate epitope, CD52g, and rapidly agglutinates and immobilizes sperm (44). Formulated into a vaginal film (ZB-06), HCA was recently tested in women in a phase I clinical trial. Not only was the antibody film safe, it effectively eliminated progressively motile sperm in cervical mucus in the post-coital test (45). Multivalent variants of HCA, which are 10–100 times more potent than the parent HCA IgG mAb, are currently being developed for potential use as second generation mAbs in contraceptive films and intravaginal rings (46). Mabs directed against other sperm antigens, originally identified as contraceptive vaccine candidates (47), are also in development.

## Use of combinations of mAbs

A combination product incorporating mAbs against various STI pathogens and contraception targets such as sperm can take advantage of the specificity and safety profile of mAbs while maintaining the breadth required for a useful MPT. Mab combinations provide a suite of products that can be applied systemically or topically in fast- or extended-release formats to cover a gamut of delivery modalities and dosage regimes. For example, combining the film products from the MB66 and ZB-06 trials as a preventive for HIV, HSV and unintended pregnancy is an exciting avenue for the future of MPTs.

## Reducing barriers to mAb MPTs

The barriers to widespread use of mAbs for infectious diseases and contraception include high cost, manufacturing challenges and limited delivery methods. Most mAbs are currently used in developed countries and current mAb production costs range between $95 and $200 /g (48). The WHO advises that costs need to decrease to <$10 /g to make mAbs feasible for use in developing countries. Advances in antibody engineering that increase antibody potency and extend serum half-life, and novel mAb manufacturing platforms and delivery methods will reduce the cost and promote the feasibility of mAb MPTs. In addition, exploration of antibody features computationally can guide antibody engineering.

### Advances in mAb engineering

Antibodies are well-studied molecules with many potential avenues for improvement. There are two main defined areas in which antibodies can be engineered: the antigen-binding fragment (Fab) and the crystallizable (constant) region (Fc) that interfaces with the immune system.

### Engineering the Fab region

In the Fab region, antigen-binding sites on two Fab arms specifically recognize distinct areas (antigenic epitopes) on molecules or portions of molecules. The Fab region can be modified to increase the strength of the interaction between antibody and antigen (affinity and avidity), and/or to enable each Fab arm to bind to a different antigen (bi-specificity).

#### Increasing affinity with directed evolution

Engineering mAbs for greater affinity to antigen enables the use of lower mAb doses to achieve similar therapeutic effects, thereby lowering the cost. Protein engineering efforts to increase the affinity of natural antibodies have made great advances in the last decade, particularly as directed evolution platforms such as phage and yeast display increase screening capabilities. These automated platforms and systematized screens of millions of variants reduce the cost and labor involved with panning for higher affinity variants. Using these techniques, MedImmune improved an existing RSV monoclonal antibody known as palivizumab, resulting in a 70-fold higher affinity for antigen and 18-fold more potent neutralization of RSV *in vitro* (49). This improved mAb, called motavizumab, was compared to its parent antibody in a non-inferiority clinical trial, where it was deemed superior to its parent antibody with a 50% relative risk reduction in medically attended infections in children (50). Furthermore, the use of machine learning to inform variant selection even further reduces costs and labor associated with generating higher affinity antibodies from a parent sequence (51, 52). For example, a deep learning approach was recently used to select more potent variants of trastuzumab, a mAb used for cancer therapy (53). Applying such efforts to known STI and contraceptive mAbs could supercharge their affinities and reduce the doses required to observe therapeutic activity.

#### Increasing avidity with multivalent mAbs

While affinity describes the strength of a one-to-one molecular interaction, avidity describes the total binding strength between antigen and antibodies which have two or more antigen binding sites. The relationship between the number of binding sites on an antibody and its avidity is not a linear multiplier due to spatial effects from the first site of binding that affect subsequent binding events. The classic analogy for avidity is Velcro, where individual interactions can be weak, but many interactions together are much stronger than the sum of the individual interactions. Thus, the addition of more binding sites can supercharge the potency of a monoclonal antibody drug, and result in lower doses for the same therapeutic value. For example, the multimerization of the HIV mAb PGDM1400 showed a 40-fold improvement in neutralization (54). Avidity effects are also important for neutralization because the strength of bivalent or multivalent interaction between antigen and antibody are such that binding is irreversible on a biologically relevant timescale (55). In addition, many Fc effector functions are dependent on avid binding and clustering of antibody such as complement deposition, which is greatly enhanced by hexamerization of IgG (55).

Multivalent antibodies are developed in two different ways. The first is to mimic natural antibody isotypes that have more than two Fab arms, such as IgA (4 Fabs), and IgM (10 Fabs). The second is to engineer novel constructs with additional Fab regions on an IgG backbone. Using scaffolds that mimic nature enables a robust safety profile, and reasonable predictions of Fc functions, while using novel constructs requires more robust characterization of the immune response since there would be no natural analog molecule to guide predictions. Some novel scaffolds attach additional Fab arms to the Fc region, potentially inhibiting Fc function due to steric hindrance, while others add additional Fc regions to supercharge oligomerization of antibody to lead to enhanced Fc receptor crosslinking (56).

One such method, widely used to increase mAb valency, is the fusion of the IgM tailpiece to an IgG1 sequence, leading to multivalent structures that efficiently recruit complement binding (56), as well as an optimized C575S variant to reduce spontaneous hexamerization (57). Another method is the HexaBody method, where the IgG heavy chain is mutated to lead to concentration-dependent oligomerization on cell surfaces (56). Currently, 20 IgM-like antibodies are in clinical development (58). Many of these candidates have had limited regulatory success due to lack of specificity and binding strength because they have not been optimized beyond the germline sequence (58). To address these shortcomings, newer multivalent mAbs are built from optimized, potent IgG sequences. For example, 33C6-IgM, a class-switched version of an HIV antibody, was prophylactically administered to rhesus macaques and resulted in protection from high dose viral challenge (59). In addition, DH1017.IgM, a neutralizing IgM against Zika virus, demonstrated a 5-fold reduction in dose required for prophylaxis in mice than its IgG counterpart (60).

#### Increasing breadth and avidity with bi- and multi-specific mAbs

Increased avidity and breadth can be achieved with bi- and multi-specific antibodies that target multiple antigenic epitopes on the same protein. IgG mAbs typically have two Fab arms that bind to the same antigen. However, the natural IgG structure can be modified so that each Fab arm binds to a different epitope. This can be a different epitope on the same protein, or an epitope on an entirely different protein. This modified structure can be helpful when targeting a single epitope does not lead to clinically efficacious antibody activity, or to increase the breadth of an antibody to achieve coverage of additional pathogen variants. In the MPT field, this can be helpful for prevention of genetically diverse pathogens, such as HIV or gonorrhea that historically have become resistant to antiviral and antibacterial drugs respectively.

These recombinant bispecific antibodies (bsAbs) are generated in two different classes—those lacking an Fc region and those containing an Fc region (61). BsAbs lacking an Fc region are often fusion proteins of antibody fragments (scFvs or Fabs) or single chain antibodies (VHHs). BsAbs containing an Fc region are more complex to synthesize, with many different possible structures. Some notable structures include a knob-into-hole structure, which looks like a typical IgG with two different Fab arms, a C terminal fusion, where a Fab region is fused onto each Fc chain, and an N terminal fusion, where there are two different antigen binding regions on each Fab arm (62). There is a significant overlap between increasing breadth and increasing multivalency because many bsAb constructs, including the N and C terminal fusions, have more than two Fab regions total. The targeting of multiple epitopes can enhance avidity, especially for sparse epitopes where binding multiple sites of the same epitope may not physically be possible. HIV is one such example, where it is estimated that only 14 HIV Env proteins are on each virion (63). In addition, the use of bsAb constructs in multivalent structures can enable a huge range of different ratios between the different Fab regions that can be tuned with transfection ratios in production.

Historically, a challenge in widespread adoption of bsAbs is quality control in production. Typical production platforms use co-transfection of two different heavy and light chains encoding two different Fab arms, but this can lead to variable expression because both the homodimer and the heterodimer can be produced, and because the expression of the four different antibody chains may not be equal (62). Purifying these mixed populations of antibody is difficult and adds additional costs to manufacturing. Fusion of antibody fragments that do not contain Fc regions partially addresses these manufacturing constraints, since a single continuous sequence can be transfected with no assembly required.

Currently, 85 bsAbs are in the clinical development pipeline, mostly for the treatment of cancer and autoimmune disease (61). An example of a potential bsAb that may be useful in an MPT product is bispecific HIV mAb, 10E8V2.0/iMab, which binds both CD4 and HIV-1 gp120. This bsAb demonstrated great breadth and potency, neutralizing 118 different pseudotyped HIV strains at an IC50 of 0.002 µg/ml (64).

#### Modeling antibody Fab parameters computationally

Biophysical models of antibodies use input parameters such as number of antibodies bound per cell, crosslinking rates, and surface antigen density to understand how binding of antibody can lead to downstream signaling (65). This modeling paradigm has been applied thoroughly in cancer immunotherapy to understand how best to deliver antibody drugs, and what affinity ranges are best for particular antigens. These insights can be extended to the fields of infectious disease and immunocontraception to understand how best to optimize antibodies for neutralization, mucus trapping, complement lysis, phagocytosis, and agglutination. Some of these methods have been applied in the field of mucus trapping, where it has been demonstrated that multiple antibodies binding to a single virion is required for trapping, and low affinity bonds between Fc and mucin are instrumental in trapping virus (66). Extending these analyses to other functions of antibodies could lead to a mechanistic understanding of what antibody parameters are important to design improved prophylactic antibodies.

### Engineering the Fc region

The Fc region governs several antibody functions. Sequences in the antibody Fc region can: (1) bind to classical Fc receptors (FcR) on immune cells such as FcγR1, FcγRIIa/b and FcγRIIIa, enabling them to mediate antibody-dependent cellular phagocytosis (ADCP) and antibody-dependent cellular cytotoxicity (ADCC), mechanisms that inactivate and clear pathogens and infected cells; (2) bind to TRIM21, a cytosolic high-affinity FcR that functions to degrade antibody-bound viruses, a process called antibody-dependent intracellular neutralization (ADIN); (3) bind to the neonatal FcR (FcRn), a cytoplasmic receptor expressed by many cell types including mucosal epithelial cells and endothelial and placental cells, that plays a role in IgG transport and recycling; and (4) bind to C1q in serum and initiate the complement cascade which leads to the lysis and clearance of pathogens, infected cells and noninfected target cells, a mechanism called complement dependent cytotoxicity (CDC). In addition, the Fc region has been implicated in trapping of sperm and pathogens in mucus (67, 68). Thus, several functions and pharmacokinetic profiles of mAbs are dependent on distinct features in the Fc region and specific interactions with various receptors and proteins in tissues, mucus and serum. Many approaches including phage display, alanine scanning mutations and structure-based design have been successful in optimizing Fc functions of mAb-based biologics.

#### Increasing mAb serum half life

Increasing the half-life of mAbs is a major consideration for MPT products. The ideal mAb would have a predictable, consistent, and long half-life in serum or mucosal secretions. Currently, most mAbs are produced with similar human IgG1 backbones; point mutations of that backbone are explored for potential enhancement of pharmacokinetic properties. The site that has received the most attention is the region that interacts with FcRn. FcRn extends the half-life of IgG by reducing lysosomal degradation in endothelial cells and immune cells, and mutations that increase the stability of the Fc:FcRn interaction can increase the half life of mAbs in serum. An early prototypical mutation to the FcRn binding site that extended IgG half-life nearly four-fold was the YTE mutation (M252Y/S254T/T256E). However, the YTE modification has lower thermal stability and lower ADCC potential compared to antibodies containing the native Fc domain. LS (Met428Leu Asn434Ser) is a currently popular Fc mutation that increases binding to FcRn and enhances circulating mAb half life several-fold (69). This mutation has been applied to many clinically relevant mAbs, including HIV bnAbs VRC01 and PGT121, where the LS mutation was found to increase half-life 4.7 and 2.9 fold, respectively (70, 71). Additional mutants, such as the quadruple mutant, Ser298Ala, Glu333Ala, lysine 334 alanine (Lys334Ala), and Asn434Ala, known as the AAAA mutant, have also been introduced to extend mAb half-life (61). For systemically delivered mAbs, these mutations can reduce the intervals between dosing and thus reduce cost. For mucosally delivered mAbs, additional characterization of pharmacokinetic profiles of these mutant Fc regions can inform variant selection, since the expressed Fc receptors may be in different proportions, and mucin-binding will also play a role in clearance of the mAb.

#### Modulating Fc receptor and C1q binding

Antibody engineering has introduced numerous strategies to optimize the ability of Fc to bind to FcRs and C1q. At least 27 Fc modifications have been described that enhance FcγR binding, and 10 modifications have been described that enhance CDC (69). The HIV prevention field is exploring the impact of such Fc modifications on HIV infection in animal models and clinical trials; it is possible that some of these modifications will boost the efficacy of mAb-based MPTs. In addition, Fc mutations that ablate Fc functions are also being explored for some applications to reduce the risk of inflammation and antigen presentation associated with ADCP and complement activation (69).

## Cost-effective mAb manufacturing platforms

Big constraints on using monoclonal antibodies as MPTs are cost and manufacturability. The cost to the end-user needs to be affordable, and large quantities of the drug product need to be produced at clinical grade. It is estimated that global demand for a mucosal MPT based in mAbs would require 2 metric tons of antibody production annually (48)*.* Innovations in mAb manufacturing in the last decade have contributed significantly to reducing costs and scaling production. Currently, mAbs can be produced in cell lines in bioreactors, and in transgenic plants.

### Mammalian cells

Mammalian cell lines, including Chinese Hamster Ovary (CHO), murine myeloma NS0, and Human Embryonic Kidney-293 (HEK293) cells, have been used to produce mAbs for clinical use. These cells are grown in suspension, continuously stirred, and perfused with fresh media. Antibody is directly secreted into the culture media, which is collected and purified, typically about 1–10 grams/L (48). A potential pitfall of the CHO and NS0 cell lines includes aberrant glycosylation, and the lack of ability to produce *α*(2–6)-sialic acid residues on mAbs (72). These cell lines can be genetically modified to affect their glycosylation to modify effector function. For example, producing antibodies with afucosylated N-glycans by inactivating the Slc35c1 and FUT8 gene in CHO cells allows for enhanced interaction with Fc receptors (61). Human cell lines do not produce aberrant glycoforms but viral contamination is a concern. Costs are highly dependent on scale of production, varying from 26 to 134 dollars per gram (73).

### Transgenic plants

Transgenic plants provide another platform for mAb production and have several advantages. These plants are transiently transfected with DNA encoding the mAb of choice, grown in green houses, then the leaves are collected and mAb is purified. Using RNAi or genome editing, the native glycosylases that encode fucosylation and xylosylation have been knocked out to obtain more human like glycoforms (74). These glycoengineered plants produce more functional mAbs, with higher ADCC activity and enhanced Fc receptor binding (74). They are quite cost-effective, reaching mg mAb per kg yields, require no cold chain, are highly scalable (48, 74). HIV mAbs have been produced at scale using plant platforms, in particular VRC01 and P2G12 (7, 75). Antibodies used in the MB66 and ZB-06 MPT clinical trials were produced in nicotiana plants and were deemed safe and effective (7, 45).

### Other systems

Other production systems, including bacteria, yeast, and insect-derived cell lines have been explored for their utility in recombinant protein production. However, the mAb glycoforms that result are different from human glycans and likely immunogenic. Glycoengineered yeast, such as *P. pastoris,* result in a more human-like glycoform profile, while maintaining a high yield around 1.6 g/L of culture. Fungi have also been investigated as a potential production platform, with yields around 24.5 g/L of culture, though glycoengineering efforts remain ongoing for this species (48).

## Optimizing mAb delivery

### Systemic administration

Most clinical mAb therapies use parenteral administration [i.e., intravenous (IV) infusions or subcutaneous (SC) injections]. The recent Antibody Mediated Prevention (AMP) trials delivered 10 mg/kg or 30 mg/kg of anti-HIV mAbs by IV infusion every 8 weeks for a total of 10 doses for HIV prevention. The advantage of IV infusions is the rapid delivery of large amounts of antibody into the systemic circulation. Pharmacokinetic studies indicated that the maximum antibody titer was achieved in blood within 8 h, and effective antibody titers persisted for 8 weeks. However, these trials also showed that only a fraction of the systemically infused antibody appeared in genital or rectal secretions where protection is needed (0.4% to 28% of serum levels) (76), indicating that systemic infusion may be too expensive and ineffective for the delivery of MPTs. Extravascular mAb injections [SC or intramuscular (IM)] are also used for some applications. Because of the limited solubility of IgG (about 100 mg/ml), and the fact that only small volumes of antibody solution can be administered via SC and IM routes (2.5 ml and 5 ml per injection site, respectively) these approaches are usually reserved for very potent antibodies. However, SC and IM administration is more convenient and less costly than IV infusions as they can be self-administered or administered by a healthcare professional and do not require hospital services.

### Topical application

The MPT field has pioneered topical delivery of mAbs. This approach empowers women and men to deliver mAbs directly to the mucosal surfaces (e.g., vaginal, rectal, and penile mucosa) where they are needed and requires much lower antibody doses (e.g., 20 mg/dose for vaginal films vs. 1,000 mg/dose for systemic immunization) (7, 45). In this section, we review strategies to incorporate mAbs into gels, solid micro or nanoparticles, liposomes, micelles, or emulsions to improve their stability and release kinetics. Employing multiple strategies in tandem is also possible and can enable the delivery of multiple mAbs and antiviral drugs with different release rates and durations.

To deliver mAbs topically, the physiology of the vaginal, rectal, and penile mucosa must be considered. Broadly, mucosal surfaces protect and lubricate the underlying epithelial layer. Vaginal mucus is a constantly regenerating 50-micron layer mostly comprised of hydrated (around 95% water) negatively charged glycoproteins (cervical mucins) at a normal pH range of 4–5. This mucus composition and volume are affected by reproductive hormones and stage of the menstrual cycle (77, 78). In contrast, the colorectum contains a firmly adherent mucus layer and a thick, loosely adherent mucus layer at a pH of 7–8 with a different proportion of mucin glycoproteins with slightly different viscoelastic properties than cervical mucus (79). Additionally, gastrointestinal conditions like ulcerative colitis and Crohn's Disease lead to increased mucus secretion and decreased adherent mucus in the colorectum, causing difficulty in delivering drugs unless they can easily cross through the mucus layer (79, 80). The penis is covered in skin, but has mucosal surfaces at the opening of the urethra and the inner foreskin (81).

#### Dissolvable films and Gel formulations

There is already a precedent for using hydrogels and dissolvable polymer matrices to prevent HSV-2 and HIV transmission through the delivery of small-molecule antiviral drugs such as tenofovir disoproxil fumarate (TDF), tenofovir alafenamide (TAF) and elvitegravir (EVG) to the vagina or rectum (82–85). The first human clinical trial to topically deliver mAbs to the vagina was the MABGEL trial which delivered three HIV mAbs, 2F5, 4E10, and 2G12 in 2.5 ml of gel. The antibodies were well tolerated and persisted in vaginal secretions for up to 8 h. Recently, mAbs have been delivered topically via hydrophilic dissolvable films. Two relevant polyvinyl alcohol (PVA)-based dissolvable films containing mAbs have recently been tested in phase 1 clinical trials: MB66, a film, delivering mAbs against HIV-1 (VRC01-N) and HSV-1/2, (HSV8-N) (7, 45), and ZB-06, a film containing an antisperm antibody (HCA) for contraception (45). The mAbs delivered in film were also well tolerated and persisted in an active state in vaginal secretions for up to 24 h.

#### Biodegradable polymer matrices

To create an effective MPT, it may be important to extend the drug release time. One well-researched sustained release strategy involves incorporating drugs into biodegradable micro or nanoparticles. The gold standard material in this context is the copolymer PLGA, which has been used in the delivery of peptides like Leuprorelin (Lupron) (86). PLGA undergoes bulk erosion through hydrolytic degradation. Over time, the drug diffuses out as PLGA breaks down into its constituent glycolic and lactic acids. The ratio of these constituents and the copolymer structure (e.g., random, block, alternating) create regions of varying hydrophobicity, directly affecting the release profile of the encapsulated drug. For instance, a higher ratio of lactic acid enables longer degradation and release profiles due to its increased hydrophobicity. A new approach integrating nanomedicine and film technology for extended drug release was recently described. An HIV integrase strand transfer inhibitor, MK-2048, used for HIV prevention, was incorporated into poly(lactic-co-glycolic acid) (PLGA) nanoparticles, and the nanoparticles were delivered in a PVA—PEG (polyethylene glycol) film to female macaques. This new delivery system introduced more drug to the vaginal mucosa, and PK studies indicated sustained release for up to 3 weeks in vaginal secretions (87). This approach could potentially be used to extend the release of MPT mAbs at mucosal surfaces. However, it is important to consider the fabrication method of PLGA particles as it typically uses organic solvents, high salt concentrations, or high energy mixing. These harsh conditions could affect some drugs' stability and encapsulation efficiency (88). Rigorous planning, design, and validation must be employed to ensure post-encapsulation activity and release profile of mAbs.

For vaginal delivery, mucoadhesive nanoparticles (MHNP) are the most studied. MHNPs bind to the mucosa through electrostatic interactions, physical entrapment, or chemical binding. MHNPs can be coated with or natively consist of a variety of positively charged, hydrophilic polymers like chitosan, or stimuli-responsive polymers like poly(methacrylic acid-co-butyl methacrylate) or poly (4-vinylphenylboronic acid) (89). Although mucoadhesion is efficient at delivering a drug to the vagina, for sustained release mucus-penetrating nanoparticles (MPNP) would be a better option due to high mucus clearance rates. Both surface charge and particle size influence the ability for particles to penetrate mucus. In contrast to mucoadhesives, nanoparticles in the range of 200–500 nm readily diffuse through major pores formed by mucins and glycoproteins in cervical vaginal mucus (CVM), whereas particles around 100 nm tend to become trapped in or slowed down by smaller channels (90).

#### Biodurable polymer matrices

A common method of delivering drugs is through physical encapsulation and diffusion through biocompatible non-degradable, or slowly biodegradable polymers. Contraceptive hormones are often delivered by intravaginal rings (IVRs), subdermal implants, and intrauterine devices (IUDs). The NuvaRing® (etonogestrel/ethinyl estradiol IVR, poly(dimethylsiloxane) (PDMS)), Nexplanon® (etonogestrel implant, poly(ethylene-vinyl acetate) (PEVA)), or Mirena® (levonorgestrel IUD, PDMS) are among the most popular and most effective contraceptive methods on the market today. Some HIV pre-exposure prophylaxis (PrEP) drugs are also delivered by IVRs made from thermoplastic polyurethane (TPU), PDMS, or ethylene-vinyl acetate (EVA) (63). Recently, more exotic materials like Poly(glycerol sebacate) urethane (PGSU) have been used to deliver both hydrophilic 4′-ethynyl-2-fluoro-2′-deoxyadenosine (EFdA) and hydrophobic levonorgestrel (LNG) at once (91) Translating these devices directly into an MPT, combining both contraceptives and anti-STI drugs, including mAb-based MPTs, might be more straightforward than any other methods, and research efforts are already underway (91–96).

Silicone elastomers (crosslinked PDMS, TPU, and PEVA) are the most commonly used biodurables in this field as they are bioinert, highly elastic, and have long therapeutic lifetimes. Though biodegradable options like poly(caprolactone) (PCL) or solid PLGA or PLA (poly-(lactic acid)) are also biocompatible and release drug, they do not have the same elastic properties (91, 94). Biodurable elastomer-based devices deliver drugs through diffusion out of the polymer matrix, instead of surface or bulk degradation. The degree of crosslinking in these elastomers is inversely proportional to their pore size, though, in general, more favorable mechanical properties (i.e., higher modulus of elasticity) occur in elastomers with a higher crosslinking ratio. Consequently, this tight mesh inhibits the diffusion of larger macromolecules like proteins or antibodies. Additionally, the delivery of hydrophilic drugs like mAbs is challenging as they do not readily diffuse through hydrophobic matrices like silicone and PEVA (96). These properties make creating a mAb delivery device from common IVR elastomers challenging. To remedy this, there has been research into hybrid silicone IVRs with biodegradable polymer “pods” or cores that deliver larger hydrophilic drugs, including mAbs, circumventing the pore size and hydrophobicity problems in single material elastomer IVRs (92–96).

#### Liposomes, micelles, and emulsions

Liposomes are vesicles made from a bilayer of amphiphilic molecules, typically phospholipids, with an aqueous core. In liposomal formulations, the drug payload can be encapsulated in the hydrophilic core, entrapped in the hydrophobic membrane, or attached to the surface. Micelles are formed from a monolayer of amphiphiles with a hydrophobic core. In contrast, reverse micelles contain a hydrophilic core and hydrophobic shell. Interestingly, reverse micelles can be packaged as a colloidal dispersion in non-polar solutions, like oils or silicone (PDMS) oils, and deliver hydrophilic drugs, like antibodies. The primary purpose of these systems is to extend drug release by protecting the encapsulated drug from degradation and from premature clearance in systemic delivery systems. Emulsions on the other hand are complex fluid suspensions constituting two or more immiscible phases, like oil and water or silicone and water, stabilized by surfactants. These surfactants are amphiphilic molecules that reduce interfacial and surface tension by adsorbing to the liquid interfaces (97).

Liposomal solutions are versatile pharmaceutical delivery systems used to deliver a wide range of drugs. In 2021, the FDA approved Pfizer-BioNTech's COVID-19 vaccine, the first liposomal mRNA vaccine (98). Liposomes are typically employed systemically to control the timing or targeting of drug release. Liposomes need to first be engulfed by cells and broken down to release their payload. Immunoliposomes are a type of liposome decorated with ligands, like antibodies, to target delivery of their encapsulated payload (98). Importantly, standard liposomes might not be appropriate for topical MPT applications unless there are stimuli-liable groups (e.g., pH, temperature) within the liposome membrane to destabilize and rupture them before being engulfed. Due to the commercial importance of liposomes in pharmaceuticals, utilizing them in the MPT space may be simpler than other options.

Common strategies for formulating liposomes are relatively simple and scalable. These amphiphilic systems self-assemble in water through agitation and evaporation of solvents, encapsulating aqueous drugs (98). There are no FDA-approved mAb-encapsulating liposomes, however. Traditional liposome fabrication methods can lead to the denaturation and instability of proteins and mAbs at hydrophobic interfaces or in organic solvents, like in nanoparticle synthesis. To improve on these problems, recent strategies like Supercritical Assisted Liposome Formation (SuperLip), utilize supercritical carbon dioxide to mix and expand a solution of ethanol and phospholipids before atomizing the solution into an aqueous antibody solution. This method precipitates uniform antibody-encapsulated liposomes, avoiding the high energy mixing used by other methods that cause antibody denaturation (99).

Micelles and emulsion-based systems offer a potential topical delivery mechanism through incorporating mAbs in creams or gels. As of now, there are no FDA-approved mAb-encapsulated micelle or emulsion-based systems, although there are FDA-approved emulsions containing peptides like cyclosporin (Gengraf and Sandimmune Neoral) which could serve as a template for incorporating mAbs instead (97). However, traditional top-down micelle and emulsion fabrication methods require high-energy mixing, which could disrupt and denature mAbs. To ensure stability, bottom-up strategies could be employed instead. Microfluidic mixing and hydrodynamic focusing or liquid antisolvent precipitation could be used to minimize the physical disruption of mAbs (100–102).

#### Use of DNA and RNA for delivery of mAbs to the female genital tract

### DNA

DNA delivery systems induce durable mAb production that can reduce production costs. One approach, recombinant adeno-associated virus (rAAV)-mediated antibody gene delivery, provides an innovative means to achieve passive immunization. In this case, rAAV vectors can accommodate large sequence inserts such as a mAb sequence, and can infect a variety of tissues but do not have the viral elements necessary for replication. rAAV-based gene sequences in cells are long lived and do not integrate into nuclear DNA. This approach has recently been used in two clinical trials: (1) Genes encoding the PG9 broadly-neutralizing mAb were introduced by rAAV1 to human volunteers via intramuscular injection; the treatment was well tolerated and durable antibody production was detected (103). (2) Genes encoding the VRC01 bNab were introduced by rAAV8 vectors to adults living with HIV; this treatment was also well tolerated and bioactive antibodies were detected in some individuals up to three years after injection. The antibodies, however, had no effect on CD4 cell count or viral load (104). An earlier study showed that delivery of AAV-vectored HIV mAb genes to the vaginal mucosa of rhesus macaques produced antibodies in vaginal secretions out to 3 months (105). Another recent study used electroporation to deliver a gene encoding the 2C7 mAb against neisseria gonorrhea to mice, and observed 60 days of serum expression of the mAb, as well as complement engagement that cleared acute and re-challenge infections over a 9 week period (106). These studies provide encouraging indications that DNA-vectored antibodies might be used for mucosal delivery of MPT antibodies.

### mRNA

mRNA delivery systems offer several advantages such as flexibility, rapid production and low cost, and the recent success of COVID-19 mRNA vaccines makes a strong case for mRNA safety and efficacy (107, 108). Direct delivery of mRNA encoded mAbs to vaginal and rectal mucosa may enable lower doses of antibody (107). When mRNA encoding bnHIV mAbs was delivered directly to the sheep vagina, doses of 750 µg mRNA led to an average of 40 µg/ml antibody in vaginal secretions over 28 days, with a maximum of 210 µg/ml (107). With mRNA delivery systems, the manufacturing challenges associated with IgA and IgM production are bypassed and the generation of bi- and tri- specific antibodies can be a question of co-delivery. This platform can be harnessed to combinatorically enhance protection. For example, an IgM hexamer that includes two separate antibodies against a pathogen could reduce the ability of a pathogen to escape by mutation of a surface protein. There are many exciting, as yet unknown, constructs that mRNA delivery can enable, exceeding the combinations possible with traditional mAb production techniques.

Self-amplifying RNA, mRNA sequences that include a viral RNA-dependent RNA polymerase complex, is further amplified after entering the cell (109). This enables lower dose vaccinations (0.01 µg for a SARS-CoV-2 vaccine candidate, compared to Moderna's conventional vaccine at 100 µg per dose) and can also enable lower doses for passive immunization efforts (109). With near thousand-fold lower doses needed, the multiplexing of antibody delivery could go extremely far, creating MPTs that include numerous antibodies per pathogen, covering multiple epitopes and variants, to avoid the development of resistant pathogens that are able to escape the antibodies. Such a product would be the ultimate mimic to nature's passive immunization via breast milk—harnessing the way genetic diversity in nature protects from infection with antibody repertoires instead of single mAbs (110).

## Conclusions

MAb-based MPTs hold exceptional promise. Their advantages include safety, efficacy and combinatorial flexibility. Disadvantages include high production costs and limits to scalability which are being addressed by new production and delivery systems. Rapid developments in the MPT field have been fueled by extraordinary dedication of scientists and support from private foundations and government institutions, but the field awaits industry involvement to usher new MPT products to market.
